# Differential inhibitory and activating NK cell receptor levels and NK/NKT-like cell functionality in chronic and recovered stages of chikungunya

**DOI:** 10.1371/journal.pone.0188342

**Published:** 2017-11-28

**Authors:** Subrat Thanapati, Mohini A. Ganu, Anuradha S. Tripathy

**Affiliations:** 1 Hepatitis Group, National Institute of Virology, Pune, Pashan, Pune, Maharashtra, India; 2 Sanjeevan Hospital, Majage Nagar, Latur, Maharashtra, India; CEA, FRANCE

## Abstract

The role of natural killer (NK; CD3^-^CD56^+^)/NKT (CD3^+^CD56^+^)-like cells in chikungunya virus (CHIKV) disease progression/recovery remains unclear. Here, we investigated the expression profiles and function of NK and NKT-like cells from 35 chronic chikungunya patients, 30 recovered individuals, and 69 controls. Percentage of NKT-like cells was low in chronic chikungunya patients. NKp30^+^, CD244^+^, DNAM-1^+^, and NKG2D^+^ NK cell percentages were also lower (MFI and/or percentage), while those of CD94^+^ and NKG2A^+^ NKT-like cells were higher (MFI and/or percentage) in chronic patients than in recovered subjects. IFN-γ and TNF-α expression on NKT-like cells was high in the chronic patients, while only IFN-γ expression on NK cells was high in the recovered individuals. Furthermore, percentage of perforin^+^NK cells was low in the chronic patients. Lower cytotoxic activity was observed in the chronic patients than in the controls. CD107a expression on NK and NKT-like cells post anti-CD94/anti-NKG2A blocking was comparable among the patients and controls. Upregulated inhibitory and downregulated activating NK receptor expressions on NK/NKT-like cells, lower perforin^+^ and CD107a^+^NK cells are likely responsible for inhibiting the NK and NKT-like cell function in the chronic stage of chikungunya. Therefore, deregulation of NKR expression might underlie CHIKV-induced chronicity.

## Introduction

The chikungunya virus (CHIKV)is a positive-sense, single-stranded RNA virus of the *Alphavirus* genus belonging to the family *Togaviridae* [1]. CHIKV belongs to the arthritogenic group of alphaviruses transmitted through the *Aedes* group of mosquitoes [2–4]. Re-emergence of chikungunya, with higher medical complications,since 2006 in several Asian and African countries, is a significant public health concern. Chikungunya outbreaks have been reported in America and the Caribbean Islands in late 2013 [5, 6]. Although chikungunya is a self-limiting disease usually resolved in acute stage, persistent joints pain lasts for several months or even years in 10–20% of patients after the initial infection [3, 7–11].

CHIKV-induced rheumatism (polyarthralgia and/or polyarthritis) is a hallmark of chronic chikungunya, which deteriorates the patient’s quality of life [12]. The chronic polyarthritis is mostly symmetricaland involves small and large joints of the hands and feet, mimicking rheumatoid arthritis (RA) [11].

Persistent joint pain is a common symptom also caused by other CHIKV-related alphaviruses such as the Sindbis (SINV), Ross River (RRV), O’nyong-nyong, and Mayaro viruses[10]. A higher percentage of CHIKV-infected individuals suffer from chronic arthralgia and chronic CHIKV disease, tending to severe economic loss as reported previously [8, 12, 13–16]. Chronic and incapacitating arthralgia and subsequent injury to the jointsare believed to occur because of viral and host immune-mediated effects. The precise role of different immune mediators in CHIKV-induced pathogenesisis less documented [17, 18].

Inefficient antiviral response of the host due to perturbation in its immune cell (natural killer [NK] cell, T cell, B cell etc.) functions could be a possible reason for virus persistence and/or chronic arthralgia. NK cells play an important role in the innate immune response whereas CD3^+^CD56^+^ NKT-like cells possess both innate and adaptive immune functions, with share characteristics of both T and NK cells. Both NK and NKT-like cells are essential in the host's first line defense against viral infections and can produce antiviral effector cytokines including IFN-γ and TNF-α upon activation [19, 20].NK/NKT-like cell function is regulated by differential engagement of NK cell surface receptors (NKRs), which are divided into activation (NKp30, NKp44, NKp46, NKG2D, and NKG2C) and inhibitory (CD158a, CD158b, KIR3DL1, CD94 and NKG2A) NKRs [21–24]. Subsets of NK and NKT-like cells are reported to be potent cytotoxic effector cells and producers of IFN-γ against hepatitis B virus (HBV) and contribute towards liver pathology during chronic HBV infection [25].Roles of NK cells in alphavirus infections are reported to be both protective and pathogenic [26–28]. Further, a mouse model study has shown that persistent CHIKV infection causes chronic musculoskeletal tissue pathology,which is controlled by adaptive immune responses [29]. Studies from our group and others have reported that NK (CD3^-^CD56^+^)/NKT(CD3^+^CD56^+^)-like cells mount an early and efficient response after chikungunya infection [30–32]. However, literature on NK/NKT-like cells in CHIKV disease progression/recovery is limited [9].

The current study examines the changes in peripheral NK and NKT-like cell phenotype and functional activity in a cross-sectional cohort consisting of chronic patients and individuals recovered from chikungunya.

## Material and methods

### Study population

This study was approved by the “Institutional Ethics Committee for Research on Humans” as per the guidelines of Indian Council of Medical Research, New Delhi. The methods were carried out in accordance with the approved guidelines. Furthermore, informed written consent was obtained from all the participants prior to inclusion in the study (guardians filled the consent form on the behalf of minor participants).One hundred thirty four individuals from Maharashtra, India, comprising of 35 patients in the chronic stage of chikungunya (mean age: 39 years; range: 12–65; gender ratio [male: female]: 0.6), 30 individuals who recovered from the disease (mean age: 43 years; range: 18–73; gender ratio: 1.1), and 69 controls (mean age: 30 years; range: 22–50; gender ratio: 1.3) were enrolled in the current study (Table 1).

**Table 1:** Characteristics of study population.

Parameters	Chronic	Recovered	Controls
Study population	n = 35	n = 30	n = 69
Gender ratio (Male: Female)	0.6	1.1	1.3
Age (years): mean (range)	39 (12–65)	43 (18–73)	30 (22–50)
Post infection months: mean (range)	86 (3–109)	24 (3–36)	NA
Anti-CHIKV IgM	Positive/Negative	Negative	Negative
Anti-CHIKV IgG	Positive	Positive	Negative

NA: Not applicable

Inclusion criteria were (1) chronic chikungunya patients: chronic persistent inflammatory polyarthritis/arthralgias lasting for more than three months following an episode of acute febrile polyarthritis and positive for chikungunya IgM/IgG antibodies [33]. Exclusion criteria for this group were the presence of RA factor, anti-CCP antibody, ANA positivity and raised serum uric acid. (2) Recovered individuals: those recovered from acute chikungunya infection and remained asymptomatic for more than 3 months were categorized as recovered. Recovered individuals were positive for chikungunya IgG antibodies.(3) Controls: individuals were negative for chikungunya IgM and IgG antibodies.

All the samples were tested for antibodies against dengue virus and only samples negative for the dengue virus were included in the current study.

### Flow cytometric analysis

#### NK/NKT-like cells and their receptors

Freshly drawn peripheral blood from the 134 individuals was stained with specific anti-human monoclonal antibodies as described previously [30, 34]. NK/NKT-like cells and expression of activation and inhibitory receptors were checked using the appropriate monoclonal antibodies following a previously described staining protocol [30, 34]. Briefly, monoclonal antibodies specific for CD56 (clone B159), CD3 (clone SK7/UCHT1),NKp30/NKp44/NKp46/CD244/CD161/NKG2D/DNAM-1(clone p30-15/p44-8/9E2/2-69/DX12/1D11/DX11) (BD Biosciences, CA, USA), CD94 (clone 131412) and NKG2A (clone 131411) (R and D Systems Inc., MN, USA) were used for phenotypic characterization of NK, NKT-like cells and NK receptors (NKRs).

#### Cytotoxicity assay

Using a flow cytometry-based assay, NK/NKT-like cells activity was evaluated in 12 samples from each group following a previously reported method [30, 35]. Peripheral blood mononuclear cells (PBMCs) were isolated as per previously described protocol [34]. K562 cells (ATCC) served as the target and PBMCs as the effector. Briefly, K562 cells were labelled with carboxyfluoresceinsuccinimidyl ester (CFSE) (Molecular probes, Invitrogen, USA) (0.2μM/10^6^ K562 cells). Subsequently, the labelled target cells were co-cultured with PBMCs at an effector to a target ratio of 10:1, and incubated for 6h at 37°C. In order to assess the target cell lysis, co-cultured cells were kept on ice with 2 μg/ml 7-AAD (BD Biosciences, CA, USA) and incubated for 5 min for labelling the DNA of the dead cells. Unlabelled target cells alone served as the control. Percentage lysis was calculated using following formula:

%specifickilling=[(%experimentaltargetdeath-%spontaneoustargetdeath)/(100-%spontaneoustargetdeath)]×100.

#### Degranulation assay

Degranulation activity quantitated based on anti-CD107a (Lamp1) expression was evaluated on the NK/NKT-like cells as reported previously [30, 33, 36]. Briefly, degranulation was induced by addition of K562 (target) into the well containing PBMCs (effector) at a effector: target:: 10:1 or phorbol 12-myristate 13-acetate (PMA) plus ionomycin and incubated at 37°C. Culture medium (RPMI + 10% FBS) alone served as negative control, while PBMCs stimulated with PMA and ionomycin served as positive control.After 1 h of stimulation, 4μg/ml of brefeldin A (Sigma, MO, USA) and 5 μg/ml of monensin sodium salt (Sigma, MO, USA) were added and the cells were incubated for a total of 6 h followed by staining of the cells with antibodies against the cell surface antigen. To assess the role of NKG2A and CD94 towards the impairment of NK/NKT-like cells functions in the chronic chikungunya patients, PBMCs isolated from chronic chikungunya patients were incubated with neutralizing antibodies against NKG2A (clone 131411)/CD94 (clone 131412) (R and D Systems Inc., MN, USA) as per the manufacturer’s instruction before performing the degranulation assay. Briefly, PBMCs were incubated with NKG2A (1 μg/10^6^ cells) or CD94 (2.5 μg/10^6^ cells) neutralizing antibody for 30 min at room temperature. Following incubation, the unbound antibodies were washed with phosphate buffer saline, and the washed PBMCs were co-incubated with the K562 cells as described previously [30].

#### Intracellular cytokines and perforin assay

Intracellular cytokine staining and perforin assays were performed as described previously [30, 33, 35]. *In vitro* stimulation of effector cells with target cells and the incubation period were the same as described above. After incubation, cells were washed and stained with antibodies against CD56 (clone B159) and CD3 (clone SK7) (BD Biosciences, CA, USA). The surface-stained cells for NK/NKT-like cells were permeabilized using BD Cytofix/Cytoperm solution (BD Bioscience, CA, USA) and then subject to intracellular cytokine staining using IFN-γ (clone 25723.11)/TNF-α (clone MAb11)/perforin (clone δG9) (BD Bioscience, CA, USA) antibodies.

Acquisition and analysis of the samples were performed in the BD FACS ARIA II using FACS DIVA software (BD Biosciences, CA, USA).

### IFN-γ ELISPOT assay

Chikungunya virus-specific IFN-γ release was studied using the ELISPOT assay as reported [30, 34]. Purification and inactivation of CHIKV were performed as previously described [37]. Briefly, the whole inactivated virus (10 μg/ml) was used for the enumeration of the virus-specific IFN-γ secreting spot. PBMCs stimulated with Phytohemagglutinin A (PHA) (10 μg/ml) (Sigma, MO, USA) and culture medium alone were used as stimuli for positive and negative controls, respectively. The IFN-γ spot-forming cells (SFCs) were counted in the ELISPOT reader (Carl Zeiss, Jena, Germany) using the KS ELISPOT software. The average number of SFCs in the negative control wells was set as cut-off level. Wells with a high background or without a PHA response were excluded. Number of SFCs in unstimulated wells was subtracted from the antigen-stimulated wells in each subject group for comparison.

### Software and statistical analysis

A non-parametric Mann-Whitney U-test (where the difference in variances < 4) and Kolmogorov-Smirnov test (where the difference in variances > 4) were carried out for intergroup comparison using the SPSS 2.0 software (SPSS Inc., IL, USA). The validity of the comparison among each pair of groups was assessed using Kruskal-Wallis test before the Mann-Whitney U-test and Kolmogorov-Smirnov test.Data were expressed as mean (range). *p* values < 0.05 were considered statistically significant.

## Results

### NK and NKT-like cell subsets in chronic and recovered stages of chikungunya

**Fig 1:**
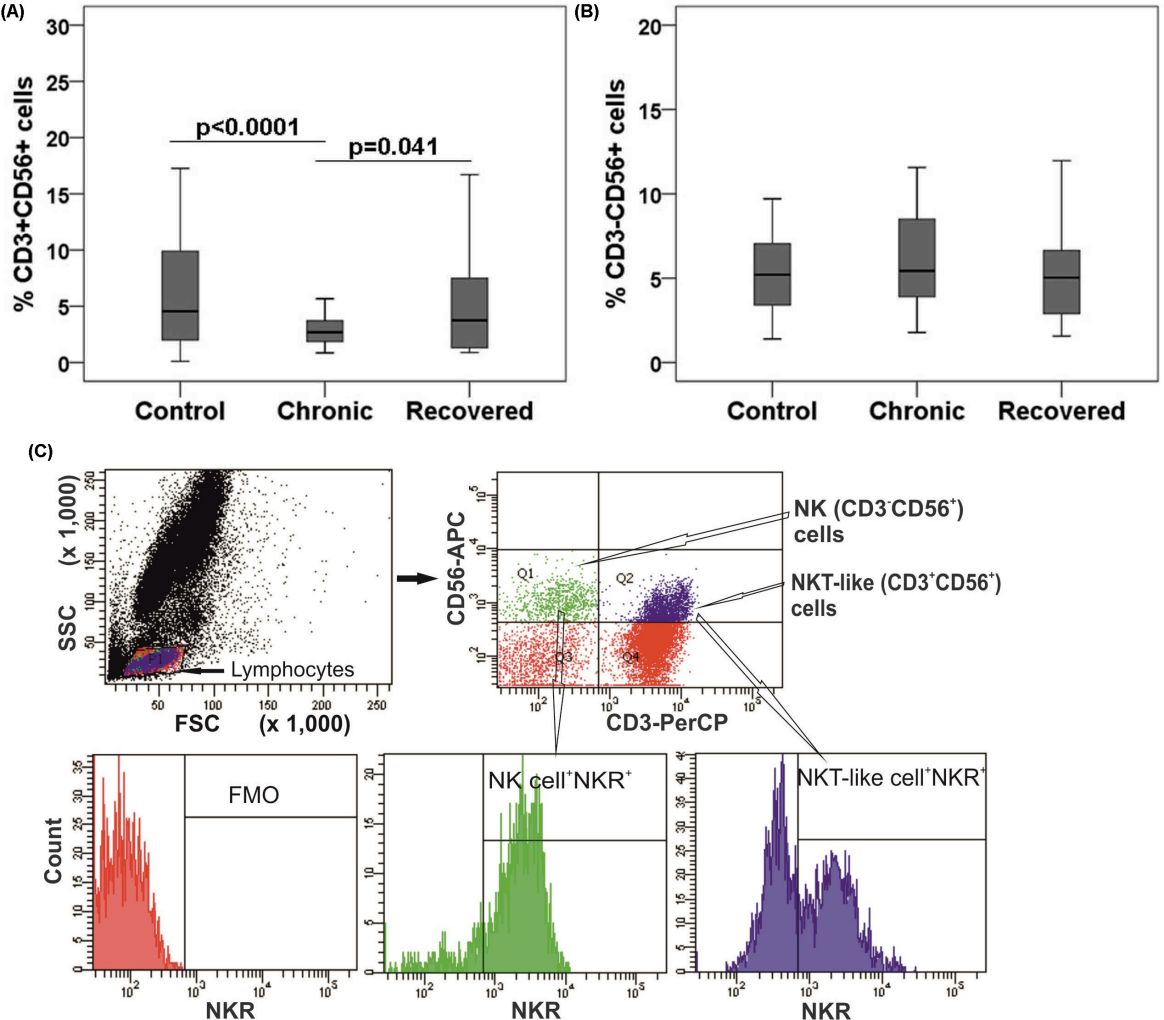
NK (CD3^-^CD56^+^) and NKT-like (CD3^+^CD56^+^) cells distribution during chikungunya. Percentages of the lymphocytes mentioned were determined from the whole blood of 35 chronic patients, 30 individuals recovered from chikungunya, and 69 controls. Box plots show the percentages of (A) NKT-like cells and (B) NK cells. Boxes represent interquartile ranges, vertical lines represent ranges and horizontal lines represent medians. Nonparametric Kolmogorov-Smirnov-test was used for intergroup comparison. *p* value <0.05 is considered significant. (C) Gating strategies used for NK, NKT-like and NK receptors^+^ (NKRs^+^) NK/NKT-like cells were same as described earlier [30]. Lymphocytes are gated in FSC vs. SSC plot, upper left quadrant (Q1) of contour plot shows NK cells (CD3^-^CD56^+^) and upper right quadrant (Q2) shows NKT-like cells (CD56^+^CD3^+^). NK/NKT-like cells were gated within the lymphocytes as per CD56 and CD3 staining pattern and NKRs were gated on NK/NKT-like cells individually. The gate for NKR expression was set as per the expression in fluorescence minus one (FMO) cells.Histogram plots represent the percentage of NKRs^+^ (NK receptors) NK/NKT-like cells.

In order to examine the phenotype of innate immune cells post acute chikungunya infection, percentages of NK and NKT-like cells were enumerated in the chronic patients and recovered individuals by flow cytometry. The percentage of NKT-like cells was lower in chronic patients than in the controls and recovered individuals (chronic: mean, 3.1[range, 0.9–15.5], vs. recovered: 5.1[0.9–18.3], and vs. controls: 6.2 (0.1–26.1; *p*<0.05in each case) (Fig 1A). Absolute count of NKT-like cells was lower in the chronic patients than in the controls (chronic: 138[67–447], controls: 150[46–492] per mm^3^ in each case). The percentage of NK cells was comparable among the three groups (Fig 1B). However, absolute count of NK cells was lower in the chronic patients than in the controls (chronic: 132[52–239] and controls: 180[101–362] per mm^3^ in each case). Gating strategy adopted for NK and NKT-like cells are represented in Fig 1C.

### Expression of NKRs on NK cells

**Fig 2:**
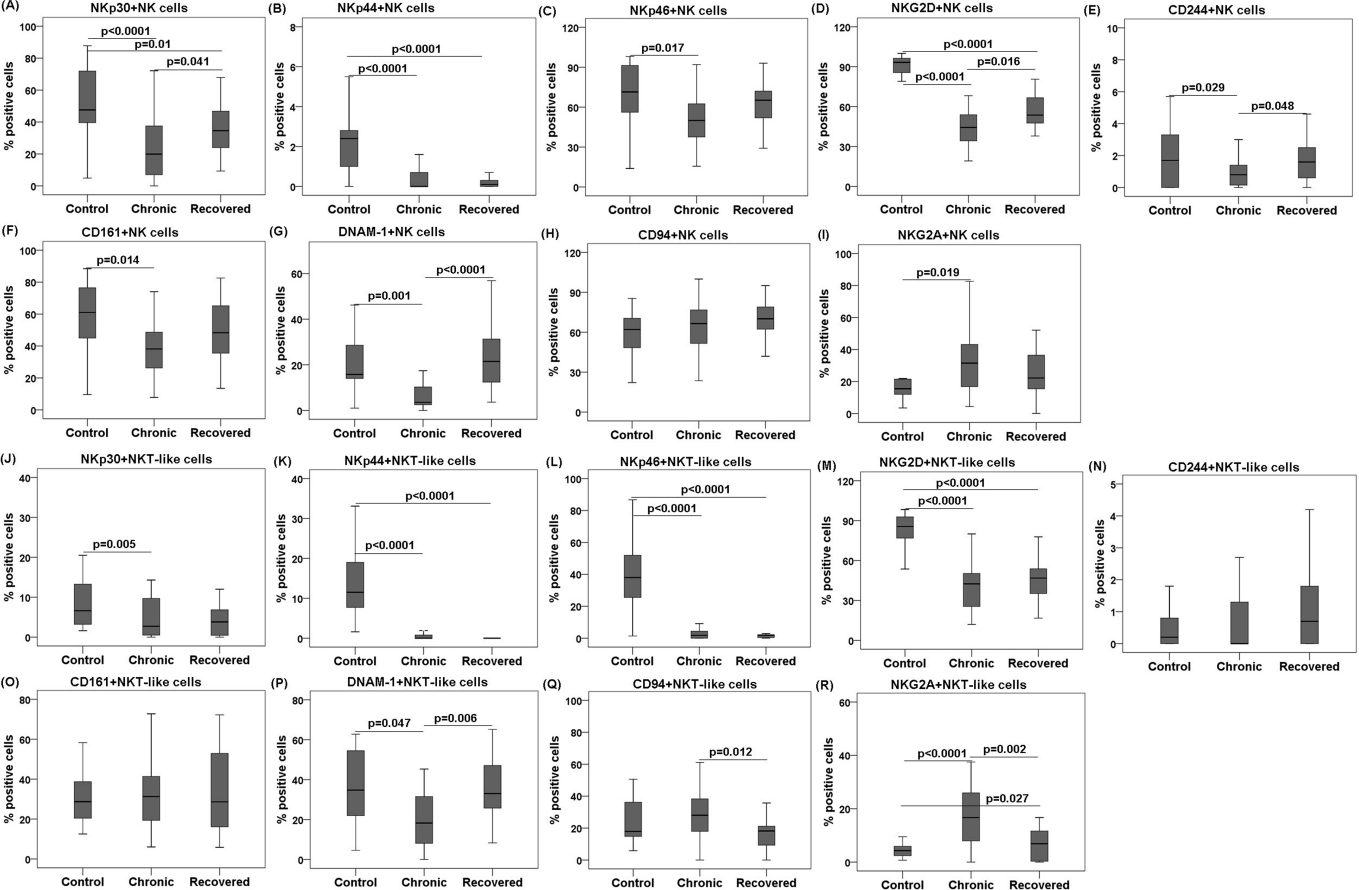
NK receptors (NKRs) expression on NK and NKT-like cells during chikungunya. Frequencies of NKRs were enumerated from the whole blood of 35 chronic patients, 30 individuals recovered from chikungunya, and 69 controls. Box plots (A−I) show percentages of NKRs^+^ NK cells as (A) NKp30, (B) NKp44, (C) NKp46, (D) NKG2D, (E) CD244, (F) CD161, (G) DNAM-1, (H) CD94 and (I) NKG2A. Box plots (J−R) show percentages of NKRs^+^ NKT-like cells as (J) NKp30, (K) NKp44, (L) NKp46, (M) NKG2D, (N) CD244, (O) CD161, (P) DNAM-1, (Q) CD94, and (R) NKG2A. Kolmogorov-Smirnov-test was used for intergroup comparison. *p* value <0.05 is considered significant.

Next, we investigated whether comparable percentages of NK cells have any influence on the expression of activation and inhibitory NKRs. Percentages of NKp30^+^, NKG2D^+^, CD244^+^, and DNAM-1^+^ NK cells were lower in chronic patients than in the recovered individuals (*p*< 0.05 in each case) (Table 2, Fig 2A, 2D, 2E and 2G). Except for CD244^+^, the mean fluorescence intensity (MFI) of NKp30^+^, NKG2D^+^, and DNAM-1^+^ NK cells were lower in the chronic patients than in the recovered individuals (Panels A, D, E, and G in S1 Fig). Lower percentages of NKp30^+^, NKp44^+^, and NKG2D^+^ NK cells were observed in the chronic and recovered groups than in the controls (*p* <0.01 in each case) (Table 2, Fig 2A, 2B and 2D).On the other hand, percentages of NKp46^+^, CD244^+^, CD161^+^, and DNAM-1^+^ NK cells were low only in the chronic patients, compared with that in the controls (*p* <0.05 in each case) (Table 2, Fig 2C, 2E, 2F and 2G). The MFI of DNAM-1^+^ NK cells was lower in chronic patients than in the controls (Panel G in S1 Fig).

**Table 2:** Percentage of NKR expression on NK/NKT-like cells.

**Types of NKRs**		**NKR**^**+**^ **CD3**^**-**^**CD56**^**+**^ **cells**	**Control** **Mean (range)**	**Chronic** **Mean (range)**	**Recovered** **Mean (range)**
Activating	NCRs	NKp30	^@^52.4(4.9−87.8)	23.7(0−72.1)	^@^38(9.3−92.3)
		NKp44	^@^2.1(0−5.5)	0.9(0−8.3)	0.3(0−1.9)
		NKp46	^@^66.4(2.2−98.1)	53.7(15.6−100)	61.9(29.1−93)
	Other cytotoxic receptors and co-receptors	NKG2D	^@^83.7(9.5−100)	46.7(19.2−95.5)	^@^56.8(37.9−80.6)
		CD244	^@^3(0.1−28.5)	1.3(0−7)	^@^3.1(0−31.3)
		CD161	^@^57.7(9.6−88.4)	41(7.8−90.5)	49.5(13.5−82.6)
		DNAM-1	^@^18.9(1−46.2)	7.3(0−27.9)	^@^23.4(3.6−68.4)
Inhibitory		CD94	58.0(12.3–85.4)	63.9(23.5−100)	67.3(2.4−95)
		NKG2A	20.4(3.5–51)	^@^30.5(4.4−82.6)	27(0.1−72.5)
**Types of NKRs**		**NKR**^**+**^ **CD3**^**+**^**CD56**^**+**^ **cells**	**Control** **Mean (range)**	**Chronic** **Mean (range)**	**Recovered** **Mean (range)**
Activating	NCRs	NKp30	^@^13.6(1.6−88.1)	8.8(0−59.6)	6.2(0−37.5)
		NKp44	^@^13.8(1.6−33.1)	0.5(0−2.8)	0.6(0−8.3)
		NKp46	^@^39.4(1.4−95.1)	4.9(0−39.7)	1.7(0−8.8)
	Other cytotoxic receptors and co-receptors	NKG2D	^@^81.3(33.8−98.3)	40.5(12−80)	45.7(16.7−77.8)
		CD244	0.5(0−3.3)	2.3(0−29.5)	1.9(0−22.6)
		CD161	31.7(12.5−72.7)	31.8(6−72.7)	33.9(5.8−72.2)
		DNAM-1	^@^36.5(4.6−62.8)	19.5(0−45.3)	^@^36.5(8.3−79.5)
Inhibitory		CD94	23.9(5.8–50.6)	^@^30.6(0−100)	19(0−58.3)
		NKG2A	5.6(0.7–28.6)	^@^19.8(0−100)	8.7(0−40.8)

^@^ symbol shows the most frequent NKR in the respective group compared with either one or both of other studied groups mentioned on the table header.

NCRs: Natural cytotoxic receptors

Nevertheless, the percentage of NKG2A^+^ NK cells was higher in the chronic patients than in the controls (*p* = 0.019) while, CD94^+^ NK cells was comparable (Table 2, Fig 2H and 2I). The percentages of CD94^+^ and NKG2A^+^ NK cells were comparable among recovered and control groups (Table 2, Fig 2H and 2I). However, the MFI of CD94^+^ NK cells was higher in chronic than those in the recovered and control groups (Panel H in S1 Fig). The MFI of NKG2A^+^ NK cells was also higher in chronic patients than in the controls (Panel I in S1 Fig).

### Expression of NKRs on NKT-like cells

Next, we investigated the changes in activation and inhibitory NKR expression on NKT-like cells. Percentage of DNAM-1^+^ NKT-like cells was lower in the chronic patients than in the recovered individuals (*p* = 0.006) (Table 2, Fig 2P). Similarly, the MFI of DNAM-1^+^ NKT-like cells was also lower in chronic patients than in the recovered individuals (Panel P in S1 Fig). Lower percentages of NKp44^+^, NKp46^+^, and NKG2D^+^ NKT-like cells were observed in the chronic and recovered groups than in the controls (*p* < 0.0001 in each case) (Table 2, Fig 2K, 2L and 2M). However, the MFI of only NKp46^+^ NKT-like cells was low in chronic and recovered groups than in the controls (Panel L in S1 Fig). On the other hand, the percentages of NKp30^+^ and DNAM-1^+^ NKT-like cells was low only in the chronic patients compared with that in the controls (*p*< 0.05 in each case) (Table 2, Fig 2J and 2P).

Higher percentages of CD94^+^ and NKG2A^+^ NKT-like cells were observed in the chronic patients than in the recovered individuals (p <0.05in each case). Percentage of NKG2A^+^ NKT-like cells was higher in the chronic and recovered groups than those in the controls (*p*<0.05 in each case) while, CD94^+^ NKT cells in the control was comparable with chronic and recovered groups (Table 2, Fig 2Q and 2R). However, the MFI of CD94^+^ and NKG2A^+^ NKT-like cells were higher in chronic than in the recovered and control groups (Panels Q and R in S1 Fig).

Gating strategies adopted for enumeration of the NKRs are described in Fig 1C.

### Impaired functionality of NK and NKT-like cells in chronic chikungunya patients

#### Altered cytotoxicity

**Fig 3:**
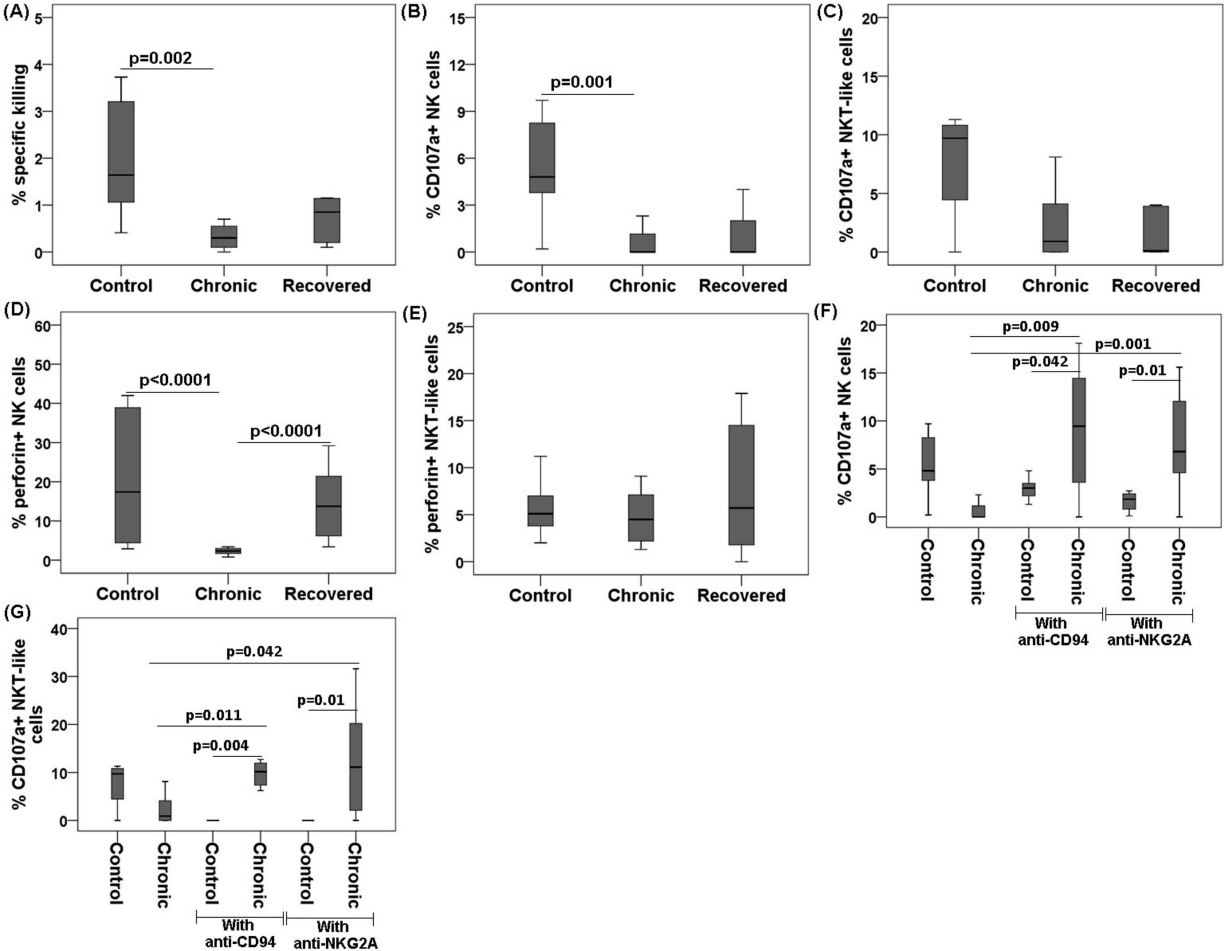
Cytotoxic and degranulation potential, and perforin expression of NK/NKT-like cells during chikungunya against target cells (K562). PBMCs (effector) were isolated from the whole blood of 12 chronic patients, 12 individuals recovered from chikungunya, and 12 controls. PBMCs were co-cultured with K562 (target) cells at effector/target ratio of 10:1 for 6 h to enumerate the percentage of CD107a on NK/NKT-like cells. Box plots show (A) % specific killing against target cells, (B) CD107a expression by NK cells, (C) CD107a expression by NKT-like cells, (D) perforin by NK cells, (E) perforin by NKT-like cells, (F) CD107a expression by NK cells in the chronic patients and controls before and after blocking with neutralization antibodies, and (G) CD107a expression by NKT-like cells in the chronic patients and controls before and after blocking with neutralization antibodies. Mann-Whitney U-test/Kolmogorov-Smirnov test was used for intergroup comparison. *p* value <0.05 is considered significant.

Since the expressions of NKRs on NK/NKT-like cells were altered in the groups studied, we performed an effector/target cell-based assay to correlate these changes with NK/NKT-like cell functionality. A lower target-cell killing was observed in the chronic patients compared with that in the controls (% specific killing: chronic 0.3 [0–0.7] vs. controls 2 [0.4–3.7], *p* = 0.002) (Fig 3A). Cytotoxicity was comparable among the recovered and control groups.

#### Altered CD107a and perforin expression

An effector/target cell-based flow cytometry assay was carried out to assess the expression of CD107a and perforin on NK/NKT-like cells. The chronic chikungunya patients had lower expressions of CD107a and perforin on the NK cells compared with the controls (CD107a: chronic 0.7 [0–3.4] vs. controls 5.4 [0.2–9.7], *p* = 0.001); perforin: chronic 2.3 [0.8–3.4] vs. controls 21.3 [2.9–42],*p*<0.0001) (Fig 3B and 3D, respectively). Similarly, the MFI of perforin^+^ NK cells was also lower in chronic patients than in the controls (Panel A in S2 Fig). The expression of these molecules was comparable among the recovered and control groups (Fig 3B–3E). Perforin^+^ NK cells were more abundant in the recovered individuals than in the chronic patients (perforin: chronic 2.3 [0.8–3.4] vs. recovered 18 [3.4–55.1], *p*<0.0001) (Fig 3D). Similarly, the MFI of perforin^+^ NK cells was also higher in recovered individuals than in the chronic patients (Panel A in S2 Fig). The percentage and MFI of perforin^+^ NKT-like cells were comparable among the studied groups (Fig 3E, Panel B in S2 Fig).

To assess the impact of inhibitory NKRs (CD94 and NKG2A) on NK/NKT-like cell function (CD107a expression), we performed another effector/target cell-based assay using neutralizing antibodies against CD94 and NKG2A in both chronic chikungunya patients and in control individuals. Blocking these molecules resulted increased the CD107a expression on NK (CD107a^+^ NK cell: chronic 0.7 [0–3.4], control with anti-CD94 2.9 [1.3–4.8] vs. chronic with anti-CD94 9.1 [0–18.1], *p* < 0.05 in each case; chronic 0.7 [0–3.4], control with anti-NKG2A 1.6 [0.1–2.7] vs. chronic with anti-NKG2A 7.8 [0–15.6], *p*<0.05in each case) and NKT-like cells (CD107a^+^ NKT-like cell: chronic 2.3 [0–8.1], control with anti-CD940 [0] vs. chronic with anti-CD94 9.8 [0–19.5], *p* < 0.05 in each case; chronic 2.3 [0–8.1], control with anti- NKG2A 0 [0] vs. chronic with anti-NKG2A 12.3 [0–31.6], *p*<0.05 in each case] in chronic patients (Fig 3F and 3G). CD107a expression on both NK and NKT-like cells post anti-CD94/anti-NKG2A blocking was comparable among the chronic patients and control individuals (Fig 3F and 3G).

### IFN-γ and tumour necrosis factor (TNF)-α expression on NK/NKT-like cells

**Fig 4:**
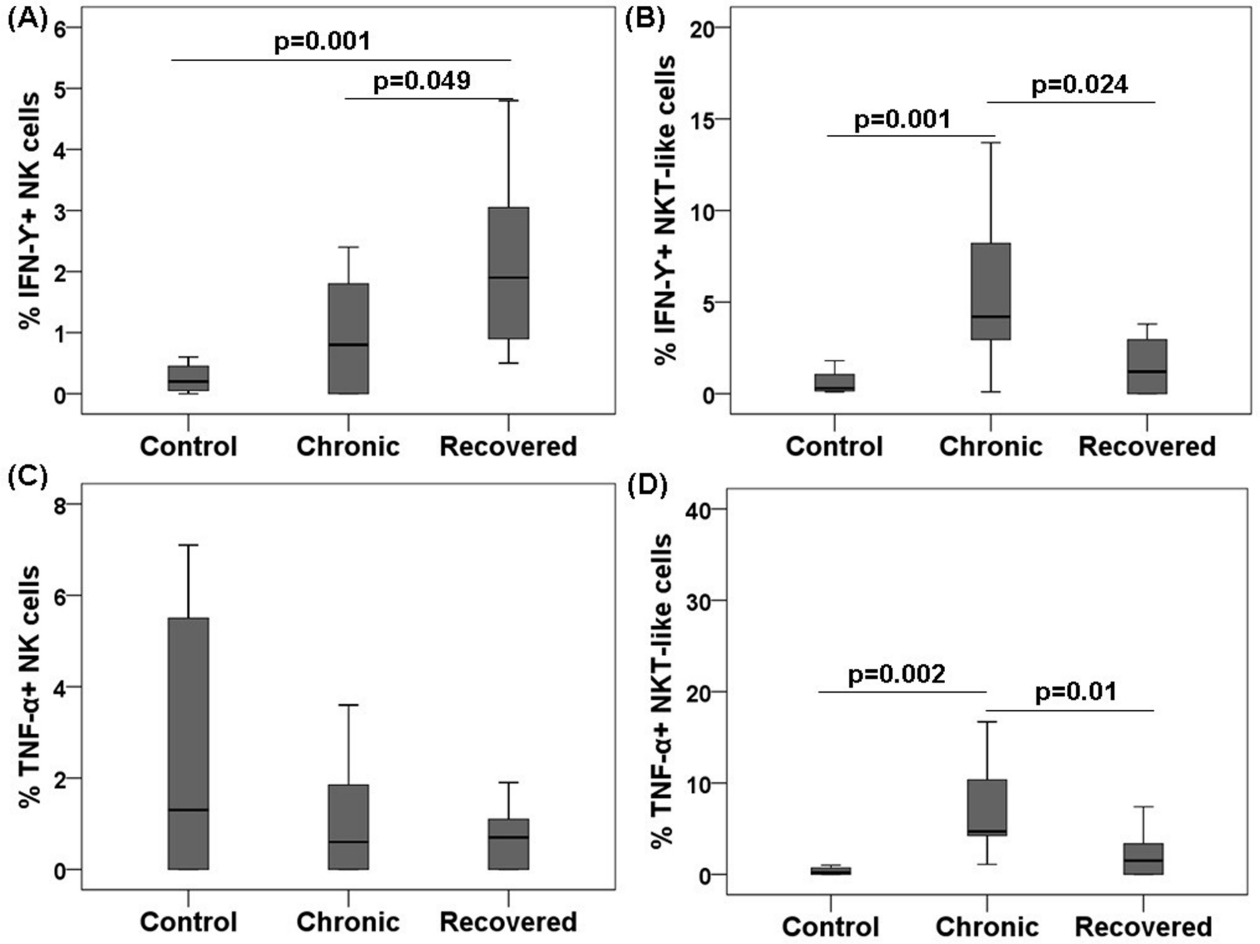
Intracellular cytokines expression of NK/NKT-like cells during chikungunya against target cells (K562). PBMCs (effector) were isolated from the whole blood of 12 chronic patients, 12 individuals recovered from chikungunya, and 12 controls. PBMCs were co-cultured with K562 (target) cells at effector/target ratio of 10:1 for 6 h. Box plots show (A) IFN-γ by NK cells, (B) IFN-γ by NKT-like cells, (C) TNF-α by NK cells, and (D) TNF-α by NKT-like cells. Mann-Whitney U-test/Kolmogorov-Smirnov test was used for intergroupcomparison. *p* value <0.05 is considered significant.

To study the effector function (cytokine production) of NK/NKT-like cells post acute chikungunya infection, we performed intracellular cytokine staining for IFN-γ and TNF-α. Higher IFN-γ expression was observed on the NK cells of the recovered individuals than in the NK cells of the chronic and control individuals (IFN-γ: recovered 2.1 [0.5–4.8] vs. chronic 1.1 [0–5.6], and vs. controls 0.3 [0–0.6], *p*<0.05in each case) (Fig 4A). However, IFN-γ expression on the NKT-like cells of chronic patients was higher than that in the controls (IFN-γ: chronic 6.2 [0.1–18.5] vs. controls 0.6 [0.1–1.8], *p* = 0.001) (Fig 4B). NKT-like cells from the chronic patients showed higher TNF-α expression compared with the other subjects(TNF-α: chronic 11.3 [2.3–75], vs. recovered 2.1 [0–7.4], and vs. controls0.4 [0–1], *p*<0.05 in each case) (Fig 4D). TNF-α expression in the NK cells was comparable among all the groups (Fig 4C). IFN-γ and TNF-α expressions in positive controls (phorbol-12-myristate-13-acetate +ionomycin, stimulated) were [IFN-γ^+^ NK cells: controls 13.5 (10–16.2), chronic 16.3 (14.2–18.3), and recovered 14.8 (13.2–17.1); IFN-γ^+^ NKT-like cells: controls 21.2 (13.2–28.1), chronic 16.7 (14.4–19), and recovered 16.3 (11.8–2)] and TNF-α [TNF-α^+^ NK cells: controls 14.5 (8.7–20.3), chronic 17.6 (16.7–18.4), and recovered 11.7 (10.8–12.5); TNF-α^+^ NKT-like cells: controls 24.9 (17.8–32), chronic 15.5 (11.8–19.2), and recovered 17.8 (15.8–19.8)] comparable among chronic and recovered groups.

### Reduced CHIKV-specific IFN-γ release in the chronic chikungunya patients

**Fig 5:**
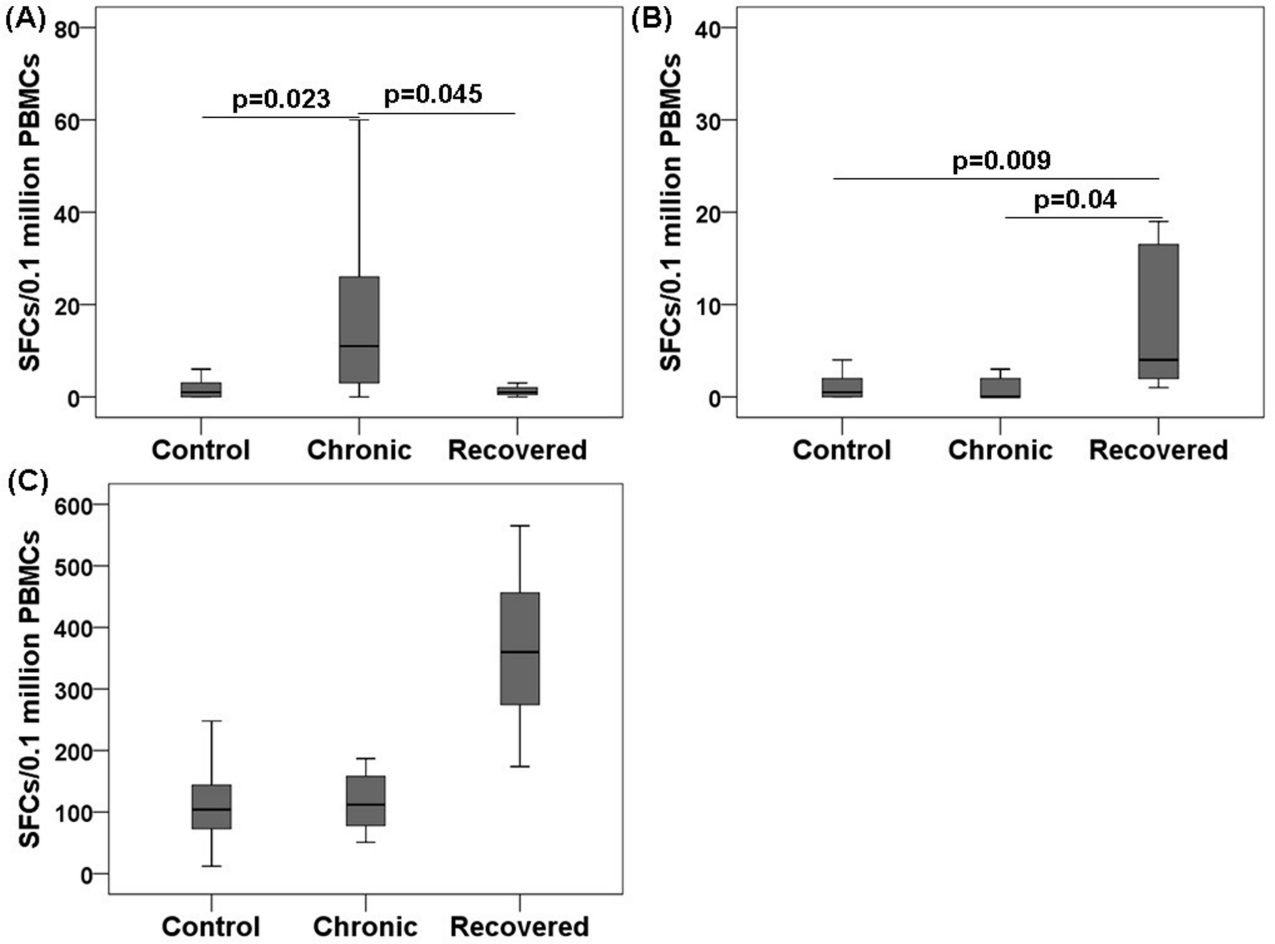
Ex vivo IFN-γ responses by ELISPOT assay during chikungunya. PBMCs from 12 chronic patients, 12 individuals recovered from chikungunya, and 18 controls were assessed using IFN-γ ELISPOT assay. Box plots show (A) IFN-γ production in negative controls (PBMCs stimulated with medium only) (B) CHIKV specific IFN-γ production (after subtraction of spots in unstimulated PBMCs well from stimulated well) and (C) IFN-γ production in positive controls (PBMCs stimulated with Phytohemagglutinin A [PHA]). Nonparametric Kolmogorov-Smirnov-test was used for intergroup comparison. *p* value <0.05 is considered significant.

To determine the CHIKV-specific IFN-γ response, we performed an enzyme-linked immunospot (ELISPOT) assay using whole CHIKV particles as antigen. IFN-γ release in the negative control (medium-stimulated cells) was higher in the chronic patients than in the control and recovered individuals (chronic 23 [0–91] vs. recovered 1 [0–8], and vs. controls 1 [0–6], *p*<0.05 in each case) (Fig 5A). In addition, CHIKV-specific IFN-γ release was higher in the individuals recovered from chikungunya compared with chronic patients and controls (recovered 14 [1–79] vs. chronic 1 [0–7], and vs. controls 1 [0–8], *p*<0.05 in each case) (Fig 5B). IFN-γ release in the positive controls (Phytohemagglutinin A [PHA]-stimulated cells) were 113(25–248), 130(51–512), and 362(174–565) in the control, chronic, and recovered groups, respectively (Fig 5C).

## Discussion

Having previously established the potential role of cytotoxic NK/NKT- like cells in influencing the pathogenesis of early CHIKV infection, here we attempted to understand the mechanisms that limit viral dissemination/establishment of chronicity in chikungunya disease. Immune effector cells, such as NK cells, have been implicated in the early control of various viral infections; hence, it was of interest to investigate the intrinsic association between deregulated NKR expression and lymphocyte function in both chronic patients and individuals recovered from chikungunya infection.

Impairment of NK cell function in the chronic patients, irrespective of NK cell percentage being comparable with the controls, may be a specific phenomenon indicating that the functional capacity per NK cell in the chronic chikungunya patients is low. Further, lower percentage of NKT-like cells in the chronic patients may be due to high turnover of NKT-like cells or due to their migration into the inflamed joints during chronic stage of chikungunya. Considering its substantial role in host’s antiviral defense, impact of lower percentage of NKT-like cells with the chronicity of CHIKV infection cannot be ruled out.

Our data showed that the expression of inhibitory receptors NKG2A and CD94were high (MFI and/or percentage) on the NK/NKT-like cells of chronic chikungunya patients. This highlights a complex interplay between NK/NKT-like cell receptors and their function with the phase of the disease. Restoration of the function (degranulation capacity) of the NK/NKT-like cells in the chronic patients following blocking with anti-CD94 or anti-NKG2A antibodies suggest investigation of their role in chikungunya associated chronicity. NKG2A has been reported to be involved in cytotoxic T lymphocyte (CTL) regulation [23, 24]. Restoration of CTL in *invitro-*expanded, NKG2A-positive melanoma cells by blocking CD94/NKG2A with a specific antibody has also been described [23, 24, 38].

Lower expression of activation receptors on NK and/or NKT-like cells in the peripheral blood of chronic patients could be attributed to infiltration of activated NK/NKT-like cells into the synovial fluid as reported previously in chronic chikungunya patients [9]. Lower percentage of activation receptor-expression on NK/NKT-like cells in the chronic patients is reminiscent of reduced surface expression of NKp30 and NKp46 in HIV-1-infected patients, another chronic viral infection [39]. Furthermore, higher expression of activation receptors NKp30, NKp44, NKp46, and CD244, and lower levels of inhibitory receptors NKG2Aand CD94 on NK cells can deliver an effective cytotoxic response, as demonstrated previously [30, 39–41]. Downregulation of activation receptors may be a possible mechanism adopted by different viruses/viral antigens to counteract NK/NKT-like cell functions. Increased percentage of NKG2D^+^NK cells in the recovered individuals compared with the chronic patients indicates their potential role in the recovery from chikungunya.

Increased levels of inflammatory mediators such as MCP-1, TNF-α and IFN-γ have been reported in many viral arthritides [42, 43]. This is corroborated by the higher percentages of TNF-α- and IFN-γ-producing NKT-like cells in the chronic patients studied here. These results also indicate their possible involvement in CHIKV-induced arthritis. NK cells are one of the earliest effector cells to respond to viral infection, where they exert their effects through IFN-γ production [44]. It is noteworthy that our current and previously published data have shown increased IFN-γ expression on the NKT-like cells of patients at different phases of chikungunya infection [30]. In a mouse model of CHIKV arthritis, levels of IFN-γ and TNF-α were reported to be elevated and were associated with arthritic inflammation [45]. Furthermore, we have previously shown higher TNF-α^+^ and IFN-γ^+^ NK-like T cells in both arthritic patient groups (chronic chikungunya arthritis and RA) [33].Higher percentages of TNF-α- and IFN-γ-expression on NKT-like cells, irrespective of lower percentage of peripheral NKT-like cells in the chronic patients of the current study is consistent with the above report and indicates probable involvement of these mediators in chikungunya chronicity. However, further studies are needed to analyse the role of IFN-γ and TNF-α producing NKT-like cells in the chronic stage of chikungunya.

The secretion of IFN-γ is a major effector function of NK cells and may have direct as well as indirect anti-viral activities. IFN-γ production has been associated with recovery from Semiliki Forest virus (SFV) infection [46], while perforin has been shown to play a crucial role in the recovery of mice from some viral infections [47]. On similar lines, IFN-γ and perforin expression on NK cells of the current recovered individuals indicate crucial role of these molecules. Contribution of IFN-γ (released in ELISPOT) towards the development of an effective adaptive immune response in recovered individuals needs exploration.

The disease affects all age groups with female preponderance [11]. Similarly, current chronic patient group contained more female than male individuals. However, statistical analyses of each assay parameter following separation by gender were comparable to the pooled results, indicating that the data were not influenced by the gender ratio.

Upregulated inhibitory and downregulated activating NKR expression might hinder the cytolytic functions of NK and NKT-like cells in the chronic stage of chikungunya, indicating deregulation of NKR expression as one of the mechanisms for defective cellular response in CHIKV-induced chronicity. NKG2D and IFN-γ expression on NK cells and CHIKV-specific IFN-γ production in the recovered individuals, compared with that in chronic patients, indicates the role of NK and/or T cell effector function in recovery. Considering the outcomes of our previous [33] and current study, we can speculate that anti-TNF-α therapy could be a treatment strategy for chronic chikungunya patients. Overall, we have shown in this study that NK cells are dysfunctional in the periphery, and have suggested a pathogenic role of NKT-like cell subsets in chronic chikungunya patients. Future longitudinal studies are required to ascertain how the phenotypic NKR deregulation and functional changes in NK/NKT-like cells modulate the persistence of chronicity in chikungunya.

## Supporting information

S1 Fig Mean fluorescence intensity (MFI) of NKRs^+^NK/NKT-like cells. The box plots show the MFI of NKRs in chronic chikungunya patients, recovered individuals, and controls. The figures (A−I) show the MFI of NKRs^+^NK cells as (A) NKp30, (B) NKp44, (C) NKp46, (D) NKG2D, (E) CD244, (F) CD161, (G) DNAM-1, (H) CD94 and (I) NKG2A. The figures (J−R) show the MFI of NKRs^+^NKT-like cells as (J) NKp30, (K) NKp44, (L) NKp46, (M) NKG2D, (N) CD244, (O) CD161, (P) DNAM-1, (Q) CD94, and (R) NKG2A. Mann–Whitney U-test/Kolmogorov-Smirnov-test was used for intergroup comparison. *p* value <0.05 is considered significant.
(TIF)

S2 Fig MFI of perforin^+^ NK/NKT-like cells. The box plots show the MFI of perforin in chronic chikungunya patients, recovered individuals, and controls. The figures show (A) MFI of perforin^+^ NK cells and (B) MFI of perforin^+^ NKT-like cells. Kolmogorov-Smirnov-test was used for intergroup comparison. p value <0.05 is considered significant.
(TIF)

S1 Table Mean fluorescence intensity of NKRs. (DOCX)
