# Supplementary figures and images for: Differential inhibitory and activating NK cell receptor levels and NK/NKT-like cell functionality in chronic and recovered stages of chikungunya
Source: PLoS One. 2017 Nov 28;12(11):e0188342. doi: 10.1371/journal.pone.0188342 (PMC5705157; doi:10.1371/journal.pone.0188342)

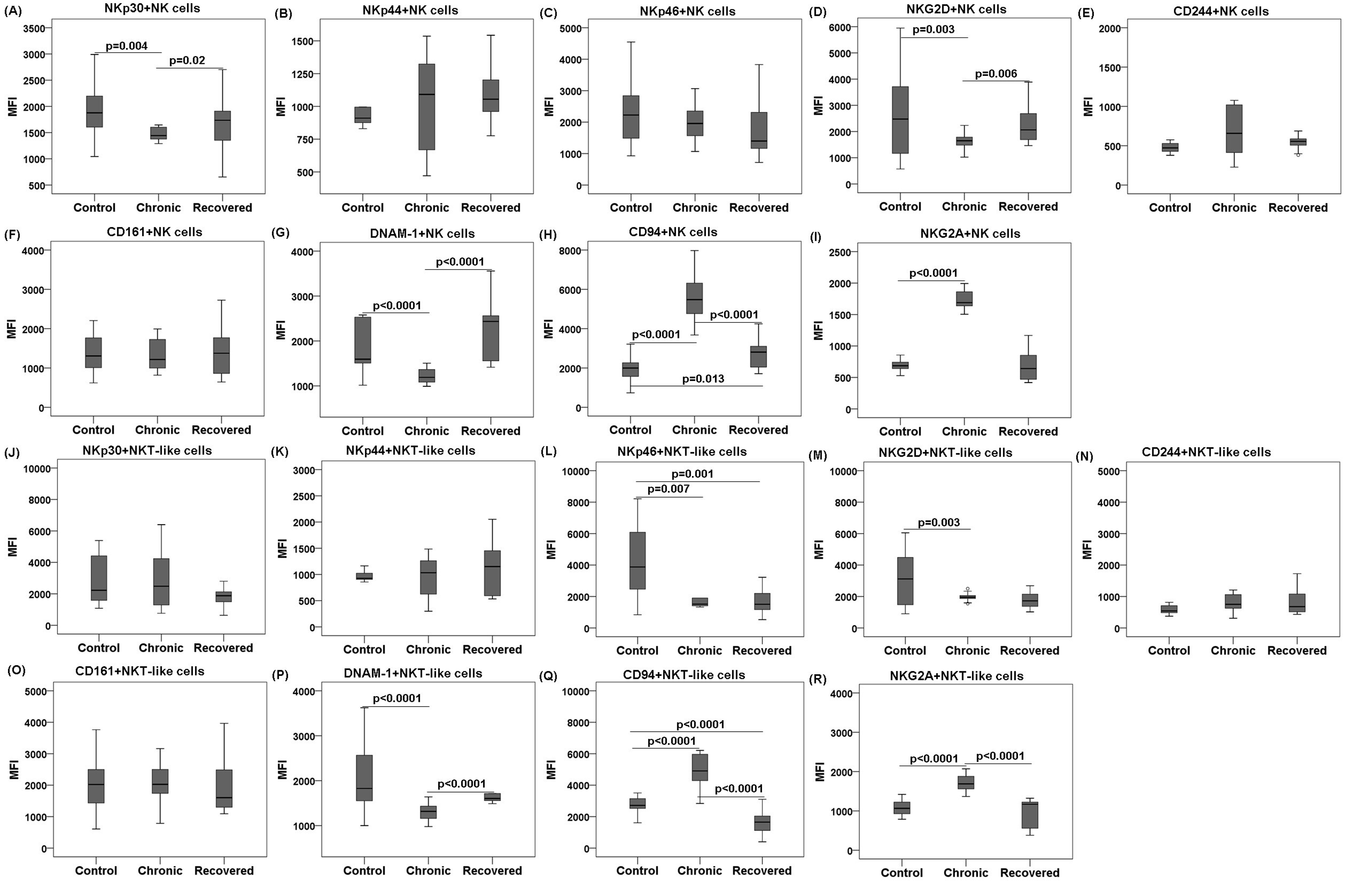

Supplement: S1 Fig — The box plots show the MFI of NKRs in chronic chikungunya patients, recovered individuals, and controls. The figures (A−I) show the MFI of NKRs+NK cells as (A) NKp30, (B) NKp44, (C) NKp46, (D) NKG2D, (E) CD244, (F) CD161, (G) DNAM-1, (H) CD94 and (I) NKG2A. The figures (J−R) show the MFI of NKRs+NKT-like cells as (J) NKp30, (K) NKp44, (L) NKp46, (M) NKG2D, (N) CD244, (O) CD161, (P) DNAM-1, (Q) CD94, and (R) NKG2A. Mann–Whitney U-test/Kolmogorov-Smirnov-test was used for intergroup comparison. p value <0.05 is considered significant. (TIF) [file pone.0188342.s001.tif]

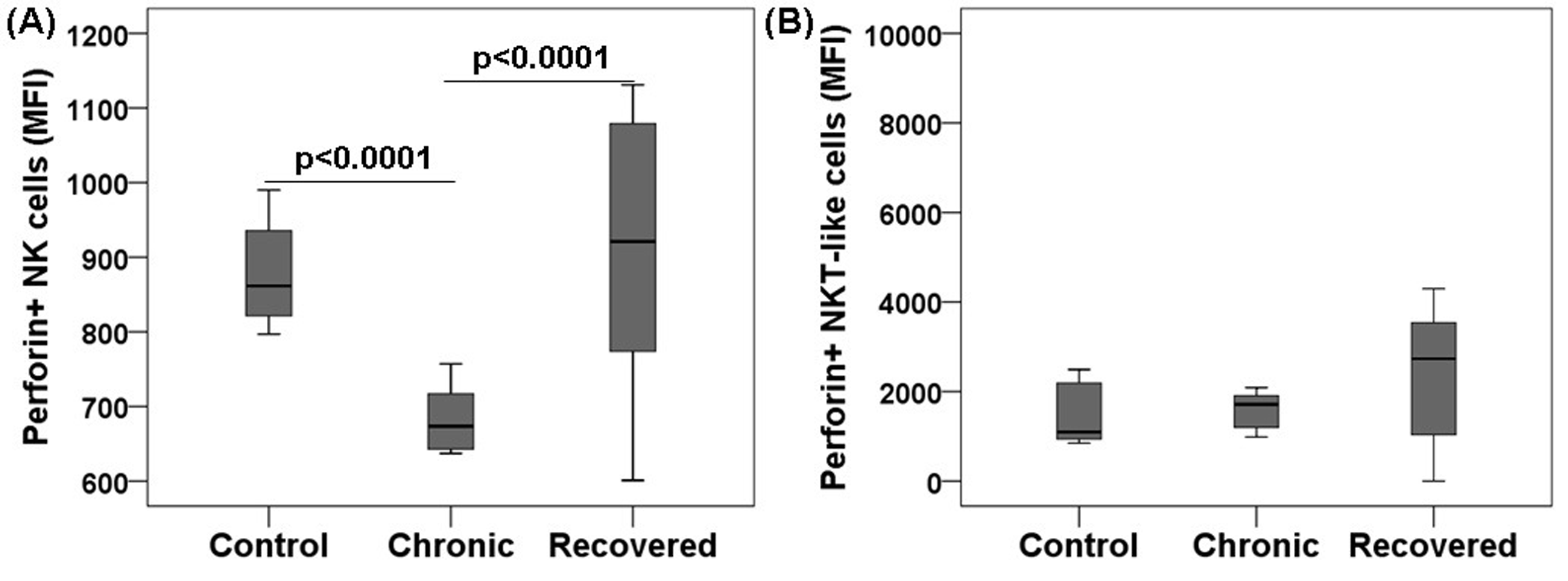

Supplement: S2 Fig — The box plots show the MFI of perforin in chronic chikungunya patients, recovered individuals, and controls. The figures show (A) MFI of perforin+ NK cells and (B) MFI of perforin+ NKT-like cells. Kolmogorov-Smirnov-test was used for intergroup comparison. p value <0.05 is considered significant. (TIF) [file pone.0188342.s002.tif]
