# Supplementary material for: Differential inhibitory and activating NK cell receptor levels and NK/NKT-like cell functionality in chronic and recovered stages of chikungunya
Source: PLoS One. 2017 Nov 28;12(11):e0188342. doi: 10.1371/journal.pone.0188342 (PMC5705157; doi:10.1371/journal.pone.0188342)
Supplement: S1 Table — (DOCX) [file pone.0188342.s003.docx]

Table. Mean fluorescence intensity of NKRs

| **Types of NKRs** | | **NKR^+^ CD3^-^CD56^+^ cells** | **Control**  **Mean (range)** | **Chronic**  **Mean (range)** | **Recovered**  **Mean (range)** |
| --- | --- | --- | --- | --- | --- |
| Activating | NCRs | NKp30 | 1952 (1044−3202) | 1567 (1290−2436) | 1734 (1054−2784) |
|  |  | NKp44 | 1016 (830−1577) | 1060 (471−1536) | 1113 (776−1543) |
|  |  | NKp46 | 2271 (928−4548) | 1981 (1067−3066) | 1772 (718−3830) |
|  | Other cytotoxic receptors and co-receptors | NKG2D | 2664 (572−5949) | 1592 (820−2234) | 2230 (1464−3887) |
|  |  | CD244 | 566 (378−1862) | 672 (228−1077) | 587 (381−1374) |
|  |  | CD161 | 1358 (620−2205) | 1326 (818−1992) | 1429 (644−3627) |
|  |  | DNAM-1 | 1871 (1016−2578) | 1220 (991−1506) | 2155 (1415−3556) |
| Inhibitory |  | CD94 | 1931 (737−3212) | 5568 (3677−7972) | 2720 (1712−4245) |
|  |  | NKG2A | 686 (529−856) | 1757 (1504−2362) | 724 (418−1475) |
| **Types of NKRs** | | **NKR^+^ CD3^+^CD56^+^ cells** | **Control**  **Mean (range)** | **Chronic**  **Mean (range)** | **Recovered**  **Mean (range)** |
| Activating | NCRs | NKp30 | 3188 (1083−8989) | 2996 (765−6401) | 2368 (636−7540) |
|  |  | NKp44 | 1091 (857−2731) | 961 (299−1485) | 1156 (534−2052) |
|  |  | NKp46 | 4259 (842−8214) | 1766 (1331−2864) | 1887 (528−6406) |
|  | Other cytotoxic receptors and co-receptors | NKG2D | 3196 (902−6050) | 1881 (1023−2498) | 2359 (1023−9081) |
|  |  | CD244 | 911 (373−4671) | 807 (308−1207) | 897 (433−2632) |
|  |  | CD161 | 2211 (608−4394) | 2153 (785−3875) | 1886 (1091−3965) |
|  |  | DNAM-1 | 1992 (1000−3623) | 1326 (979−1986) | 1817 (1489−2652) |
| Inhibitory |  | CD94 | 2792 (1353−4600) | 5042 (2837−9477) | 1653 (401−3102) |
|  |  | NKG2A | 1095 (788−2009) | 1756 (1367−2749) | 1089 (380−3703) |
